# Development and Characterization of Polymeric Films Loaded with Terbinafine for Fungal Infection Treatment

**DOI:** 10.3390/polym17081004

**Published:** 2025-04-08

**Authors:** Gabriela Biliuta, Simona Petronela Gherman, Raluca Ioana Baron, Alexandra Bargan, Lacramioara Ochiuz, Cristina Gabriela Tuchilus, Adrian Florin Spac, Daniela Elena Zavastin

**Affiliations:** 1 Polyaddition and Photochemistry Department, “Petru Poni” Institute of Macromolecular Chemistry of Romanian Academy, 41A Gr. Ghica-Voda Alley, 700487 Iasi, Romania; biliuta.gabriela@icmpp.ro (G.B.); baron.raluca@icmpp.ro (R.I.B.); 2 Faculty of Pharmacy, “Grigore T. Popa” University of Medicine and Pharmacy Iasi, 16th University Str., 700115 Iasi, Romania; lacramioara.ochiuz@umfiasi.ro (L.O.); adrian.spac@umfiasi.ro (A.F.S.); daniela.zavastin@umfiasi.ro (D.E.Z.); 3 Department of Inorganic Polymers, “Petru Poni” Institute of Macromolecular Chemistry of Romanian Academy, 41A Gr. Ghica-Voda Alley, 700487 Iasi, Romania; anistor@icmpp.ro; 4 Faculty of Medicine, “Grigore T. Popa” University of Medicine and Pharmacy Iasi, 16th University Str., 700115 Iasi, Romania; cristina.tuchilus@umfiasi.ro

**Keywords:** terbinafine hydrochloride, fungal infection, antifungal activity, dermatophytosis, micellar solubilization, bioadhesion, the release kinetics

## Abstract

Topical approaches to dermatophytosis have the advantage of targeted therapy and minimal side effects and are patient-friendly. The present study focused on obtaining thin, flexible, and transparent bioadhesive polymeric films loaded with terbinafine hydrochloride (TH), in order to be administered to the skin affected by fungal infection. Polymeric films based on pullulan (P), oxidized pullulan (T-OP), sodium carboxymethylcellulose (NaCMC), and glycerin were obtained by the casting and evaporation technique, and the solubility of the drug was significantly increased by micellar solubilization with Tween-80, thus avoiding the use of organic solvents. Physico-chemical characterization through the FTIR technique and EDX analysis indicates the absence of strong interactions between the drug and the polymer, and the loading efficiency highlights the uniform distribution of the drug. The mechanical properties, bioadhesion, contact angle, and water sorption capacity highlight optimal adhesion parameters on the skin. In vitro studies indicate a prolonged drug release, in the first 300 min, of 80% and 60% for F2_TH and F1_TH, respectively, and the release kinetics follow the Weibull model. Significant antifungal activity was obtained for both polymeric films. The biocompatibility of the ingredients, the gentle technique for obtaining the films, and the results obtained from their analysis represent promise for their applicability in topical antifungal treatment.

## 1. Introduction

Dermatophytosis, or tinea, is a condition of the skin and its appendages (hair, nails), which occurs in response to a fungal infection of the genera *Tricophyton*, *Epidermophyton*, and *Microsporum* and causes *Tinea corporis*, *Tinea pedis*, *Tinea ungium* and *Tinea cruris* [1,2]. Dermatophytosis can be managed with topical antifungal medication, the most prescribed being the azole antifungal class, but this can cause a series of anaphylactic reactions [3]. A very effective drug in antifungal therapy is terbinafine hydrochloride (TH), which has a minimum fungal concentration of 0.003–0.006 µg/mL and a minimum inhibitory concentration of 0.001–0.01 µg/mL [3,4,5]. Terbinafine is a broad-spectrum antifungal, which belongs to the class of allylamines and acts by inhibiting the enzyme squalene epoxidase, being the most used in superficial fungal infections, with both systemic and topical administration. From a structural perspective, the drug is a tertiary amine with a cationic structure; it is found in the form of a hydrochloride salt; it has a lipophilic character (log P 3.3); and according to the Biopharmaceutics Classification System (BCS) classification, it belongs to class II drugs [6]. The poor solubility of TH results in low absorption, with a bioavailability after metabolization of only 40%, which means limited effectiveness of oral medication [7]. Oral administration requires a high dose of drug to reach the minimum fungal concentration, but this increases the risk of liver and kidney toxicity [7,8]. In this context, topical administration of TH represents the most favorable treatment modality for fungal infections. In order to increase the bioavailability of drugs, increasing solubility must be considered, and a feasible method is that of micellar solubilization with surfactants [9]. In aqueous solutions, surfactants, after critical micelle concentration, self-assemble into micelles and capture the drug, lowering surface tension. The most commonly used surfactants to solubilize drugs are polysorbates, polyoxy ethylated castor oil, poly-oxy ethylated glycerides, sodium dodecyl sulfate, sodium dodecylbenzene sulfonate, lauryl macro glycerides, and mono- and di-fatty acid esters of low-molecular-weight polyethylene glycols [10]. Nonionic surfactants, such as Tween-80, are biocompatible, have low toxicity, protect drugs from enzymatic hydrolysis, prevent protein aggregation, and due to their nanometric size and interaction with lipids in the stratum corneum of the skin can penetrate the tissue, increasing drug permeability and finally increasing bioavailability [11,12,13].

Once the drug’s solubility is increased, it can be targeted and precisely administered at an optimal concentration, right at the site affected by the fungal infection, thus preventing the drug’s toxicity by avoiding the first hepatic passage and eliminating absorption problems from the gastrointestinal tract [5,7,14].

For a precise administration of the dose of antifungal drug to the affected site, polymeric films are preferred because, in addition to dosing precision, they adhere to the skin and cause controlled release of the drug. At the same time, they have the ability to maintain the concentration of the drug in the therapeutic window, thus having maximum efficiency and minimum toxicity, and they also show compliance for the patient and have an esthetic appearance [15]. Recent studies have shown that polymeric films loaded with drugs from different antifungal classes are an effective and simple alternative for the treatment of mycoses that affect both mucous membranes and the skin [16,17,18,19]. The nature of polymers has a key role in antifungal treatment, as the functional groups on the polymer surface influence drug loading capacity and their release behavior, specificity and safety [20]. In recent years, pullulan (P) has become one of the most used homopolysaccharide polymers, due to its versatile structure, which enables grafting with different functional groups, especially via oxidation reactions with various selective oxidizing agents [21]. The most well-known selective oxidation method for P involves the use of the nitroxyl radical TEMPO. This process employs the stable radical TEMPO alongside sodium hypochlorite and sodium bromide to selectively oxidize the primary hydroxyl groups linked to C6 of the anhydroglycosidic unit into carboxylic groups [22]. This reaction induces novel properties for the oxidized pullulan (T-OP) that set it apart from unmodified pullulan. The structural, thermal and oxidative stability of pullulan is given by its inability to form free radicals, due to the saturation of the double bonds in the glucose units, by hydrogenation. Furthermore, the increased solubility in water, high adhesion on skins, film-forming capacity, low oxygen permeability, non-toxicity, and non-mutagenicity make pullulan an ideal candidate for the pharmaceutical industry [23,24]. In certain situations, enhancing the hydrophilicity of native pullulan is often required. Chemical functionalization can achieve this by incorporating various functional groups, such as carboxylic groups. The inclusion of ionic groups facilitates the acquisition of polymer solutions with diverse characteristics and viscosities due to the polyelectrolyte swelling of macromolecules. In addition, oxidized pullulan has some distinct advantages that make it particularly useful in drug delivery systems, due to its enhanced biocompatibility, biodegradability, tunable properties, and functional groups that can be used to optimize drug encapsulation, release, and targeting. Its increased hydrophilicity, mucoadhesiveness, and potential for functionalization make it especially suitable for oral, mucosal, and controlled-release drug delivery applications [25].

The remarkable properties of sodium carboxymethylcellulose (NaCMC), a hydrophilic anionic polymer, capable of forming films [26], that shows excellent biocompatibility, low cytotoxicity, biodegradability and cell viability [27], have made this polymer the ideal candidate, along with pullulan, for obtaining polymeric films used in clinical applications. The association of pullulan with NaCMC forms a polymer matrix with superior properties, which causes a delayed release of the drug, especially if it has a cationic structure [28], as is the case of TH.

The aim of this work was to increase the solubility of TH by micellar solubilization with Tween-80 and subsequently to include TH in a polymer matrix with superior biological properties that adheres to the tissue long enough for the drug to be released in a controlled manner and that the optimum concentration be maintained until the polymer film is replaced.

## 2. Materials and Methods

### 2.1. Materials

Pullulan (TCI Europe, Belgium, Mn ~3 × 10^5^ g/mol by SEC), sodium carboxymethyl cellulose (Sigma Aldrich, average MW 250.000 Da, degree of substitution 0.9), 2,2,6,6-tetramethyl-1-piperidine-1-oxyl radical (TEMPO, Sigma Aldrich, St. Louis, MO, USA), sodium hypochlorite (NaClO, 15% chlorite, Chemical Company, Iași, Romania), sodium bromide (99% Alfa Cesar), glycerol (Sigma Aldrich), Tween-80 (Merck Co., Rahway, NJ, USA) and terbinafine hydrochloride with MW 327.89 (Sigma Aldrich) were used as received, without further purification. Water used in film preparation was obtained from the milli-Q water purification system (Millipore Corporation, Burlington, MA, USA)

### 2.2. Methods

#### 2.2.1. Preparation of Phosphate Buffer pH 7.4

The buffer solution (pH 7.4) was made according to the indications of Chapter 4.1.3. from European Pharmacopoeia (6th edition) [29] and simulates the specific pH of skin areas, sensitive to fungal infections, which have increased humidity [30].

#### 2.2.2. Preparation of Oxidized Pullulan (T-OP)

TEMPO-mediated oxidation of P (T-OP): P (2.5 g) was dissolved in 70 mL of distilled water under vigorous stirring. TEMPO (0.2 mmol/g P) and NaBr (2 mmol/g P) were then introduced in the reaction mixture. Subsequently, a NaClO solution (ca. 15% active chlorine, 20 mmol/g P) was added to the reaction mixture while stirring continuously. The pH of the solution was carefully maintained at about 10 by adding 2M NaOH solution. The reaction was carried out for 5 h at room temperature (RT); after that, it was quenched by adding a few drops of ethanol. The T-OP was purified by dialysis for 7 days and dried by lyophilisation.

#### 2.2.3. Preparation of Films

The films were prepared by casting methods. In a first step, the TH was dissolved in a Tween 80 solution to a final concentration of 2%. P (0.4 g) or T-OP (0.4 g) and NaCMC (0.16 g) were dissolved in 10 g of 2% Tween 80 solution of TH. Then, 0.4 g of glycerol was added to the mixture and samples were stirred for 24 h at room temperature. For the control films, the process was similar, with the difference that 10 g of Tween 80 was used. All formulations of the films are shown in Table 1. The polymer solutions were transferred onto the polystyrene plates, with a diameter of 5 cm, and then dehydrated at room temperature and atmospheric pressure under a glass funnel for 5 days. A uniform thickness was maintained for all films in the study (~0.4 mm). All the films were homogeneous with apparently smooth surfaces (Figure S1, Supporting Information).

### 2.3. The Physical–Chemistry Analysis of Polymer Films

#### 2.3.1. Development of a Spectrophotometric Method

A stock solution of TH of concentration 100 µg/mL was prepared by dissolving TH in hydroalcoholic medium (volume ratio water/ethyl alcohol 70:30). From the stock solution of TH by dilution with hydroalcoholic medium, a series of eight working solutions with concentrations between 5 and 50 μg/mL were obtained, with the experiment performed in triplicate. The average absorbance values were used to plot the calibration curve, and the data were statistically processed with the ANOVA test [31]. The absorbance of the above solutions was measured using a UV-VIS spectrophotometer (SPECORD 210 PLUS-223F2042C, Jena, Germany) against a blank sample that contained hydroalcoholic medium [32].

#### 2.3.2. Solubility Study

TH has limited solubility in water, so we sought to increase its solubility by the micellar solubilization method, with a nonionic surfactant, Tween-80. For this purpose, five aqueous solutions of Tween-80 were prepared with concentrations of 2, 4, 6, 8, and 10%, over which TH was added, at a concentration of 1%. The samples were stirred on a magnetic plate at room temperature for 24 h, then centrifuged and filtered on Whatman filter paper. The obtained supernatant was analyzed spectrophotometrically, to determine the concentration of solubilized TH.

#### 2.3.3. Estimation of Drug Loading and Entrapment Efficiency

From each polymer film loaded with TH, three samples were taken from different places, over which phosphate-buffer solution (pH 7.4) was added. In order to release TH from the polymer network, the samples were placed in the ultrasonic bath for 60 min, then centrifuged for 15 min at 3500 rpm and filtered through Whatman filter paper. The supernatant was diluted to one of the concentrations on the calibration curve and analyzed quantitatively, using the developed spectrophotometric method. Following the spectral analysis, the amount of drug loaded in the polymer films was established. The formulas used for calculation of entrapment efficiency (EE) and loading capacity (LC) are as follows [33]:

(1) $$EE,\;\%=\left(\frac{m_{TH}^{exp}}{m_{TH}^{th}}\right)\cdot 100$$

EE—entrapment efficiency, %;
$m_{TH}^{exp}$—amount of TH loaded into the polymeric film; mg;
$m_{TH}^{th}$—amount of TH that was added into the polymeric film; mg.

(2) $$\mathrm{LC},\%=\left(\frac{m_{TH}^{exp}}{m_{polymeric\;film}}\right)\cdot 100$$

LC—loading capacity of TH in polymeric films;
$m_{TH}^{exp}$—amount of TH loaded into the polymeric film; mg;
m_polymeric film_—mass of the polymeric film; mg.

#### 2.3.4. Attenuated Total Reflection Fourier Transform IR (ATR-FTIR) Spectroscopy

ATR-FTIR spectra of the films were acquired using an IRAffinity-1S spectrometer (manufactured by Shimadzu Corp., Kyoto, Japan) in Attenuated Total Reflection (ATR) infrared mode (Shimadzu Corp., Kyoto, Japan). Scanning was performed with a resolution of 4 cm^−1^ and the range covered was from 4000 cm^−1^ to 500 cm^−1^. The spectra of all samples were collected using transmission mode scanning.

#### 2.3.5. Zeta-Potential Measurements

Zeta potential (ζ) was measured using a dynamic light scattering technique (Zetasizer model Nano ZS, Malvern Instruments, Malvern, UK) with a red laser of 633 nm (He/Ne). Zeta potential (ζ) was calculated from the electrophoretic mobility (μ) determined at 25 °C. For kα >> 1 (k—Debye–Hűckel parameter and α—particle radius), the Smoluchowski relationship was used, as shown in Equation (3) [34]:

(3) $$\xi =\frac{\eta \mu }{\varepsilon }$$

where

η—viscosity;
ε—dielectric constant.

#### 2.3.6. Energy-Dispersive X-Ray (EDX)

To investigate the surface and fracture morphological features, as well as the elemental composition of the films, a Verios G4 UC scanning electron microscope (Thermo Fisher Scientific, Hillsboro, OR, USA) equipped with an energy-dispersive spectrometer (EDS, EDAX Octane Elite) was used at an accelerating voltage of 5 kV.

#### 2.3.7. Mechanical Tests

Films composed of P and T-OP underwent tensile testing at room temperature utilizing the Shimadzu Testing Machine (EZ-LX/EZSX Series, Kyoto, Japan). The films exhibited a dumbbell-like shape, measuring 50 mm × 8.5 mm × 4 mm. Three distinct tests were conducted on each sample at a rate of 1 mm/min, and the average result obtained was considered. The stress–strain curves were used to directly calculate the elongation at break and tensile strength values. The elastic modulus was determined by analyzing the slope obtained from the stress–strain curve in the linear area [35,36].

#### 2.3.8. Contact Angle Determination

Statistic contact angle (SCA) measurements were performed at room temperature (~22 °C) and constant humidity (31%) via the sessile drop method using a KSV CAM 101 goniometer equipped with a special optical system and a charge-coupled device (CCD) camera. A drop of double distilled water (~1 μL) was placed on film surfaces with a Hamilton syringe. Five measurements were managed on different parts of the films, and the highest error in the contact angle measurement did not surpass 2%.

#### 2.3.9. Dynamic Vapors Sorption Measurements

The behavior of the films, in the presence of moisture, was studied by analyzing their water vapor sorption capacity in dynamic regime (DVS) using a fully automated gravimetric equipment IGAsorp made by Hiden Analytical (Warrington, UK). The ultrasensitive microbalance which measures the weight change as the humidity is modified in the sample chamber at a constant temperature represents the most important part of this equipment. The determinations were directed by a user-friendly software package. The sample was placed in a special container; then, it was dried at 25 °C in flowing nitrogen (250 mL/min) until its weight was in equilibrium at a relative humidity (RH), less than 1%, and then the isotherm was recorded. The RH was gradually increased from 0 to 90%, in 10% humidity steps, with every step having a pre-established equilibrium time between 60 and 90 min, and the sorption equilibrium was obtained for each step. The RH decreased and desorption curves were registered [37].

#### 2.3.10. Bioadhesive Properties

Using the Brookfield Texture PRO CT3(R) texture analyzer (Brookfield Engineering Laboratories Inc., Middleboro, MA, USA), the force needed to separate the polymeric film from a mucosal surface was used as a measure of how well the bioadhesion worked. A chicken skin mucosa served as the model membrane, while a phosphate-buffer solution at pH 6.8 and 37 °C was utilized as the moistening fluid. The freshly collected chicken skin mucosa was preserved in phosphate buffer at pH 6.8 and maintained at 4 °C. The lower end of a cylindrical probe (TA5; diameter 10 mm) was glued with a film piece (1 cm^2^), and the cleaned chicken skin mucosa was stuck to the TA-MA fixture kit immersed in phosphate buffer pH 6.8 and thermostated at 37 °C. The skin and the sample were maintained in contact for 120 s with a force of 1 N applied during this duration. After 120 min, the sample was moved upward at a rate of 0.5 mm/s until complete separation of the surfaces was achieved, at which point the mucoadhesive force was quantified using Texture Pro CT V1.9 Software. Three films from each formulation were evaluated, and the mean of the three determinations was utilized to assess adhesiveness.

#### 2.3.11. In Vitro Drug Release

The study of TH release from the polymeric films was performed using the dissolution vessel of the USP test apparatus II, in phosphate-buffer solution (pH 7.4) [29]. The release study was carried out at 37 °C ± 0.5 and the stirring paddle was rotated at a speed of 50 rpm [38]. The withdrawn volume was replaced with the same fresh volume of the phosphate buffer, at various time intervals for 24 h, and after adequate dilution with the dissolution medium, the concentration of the released drug was determined by the spectrophotometric method. The amount of drug released was calculated by using the calibration curve of terbinafine, in terms of % release (Pr) as shown below [39]:

(4) $$P_{r},\%=\frac{c_{r}}{c_{l}}\cdot 100$$

where

c_r_—concentration of the released drug, µg/mL;
c_l_—concentration of the loaded drug, µg/mL.

Experiments were repeated three times and the results were expressed as mean values ± SD.

#### 2.3.12. Analysis of In Vitro Drug Release Kinetics

In order to understand the release profile and establish the diffusion mechanism from the developed polymer matrices, six mathematical models of release kinetics from porous and non-porous systems were used. Models were applied for zero-order kinetics (the ideal case, with constant release in the body and low side effects), first-order kinetics (the release rate depends on the drug concentration and is specific to the release of drugs from porous matrices), the Higuchi model (the drug release occurs by diffusion), the Korsmeyer–Peppas model (describes the Fickian and non-Fickian release of drugs from the polymeric matrix), the Weibull model (this model is used for different processes of dissolution and for comparison of the release profiles of drug from matrix of delivery), and the Baker Lonsdale model (this model is used in general for linearization of release data from several formulations) [33,40].

The equations corresponding to the six models used in the study were as follows:

(5) $$\mathrm{Zero}\text{-}\mathrm{order}\mathrm{release}\;\mathrm{model}:\;F_{t}=F_{0}+k_{0}t$$

(6) $$\mathrm{First}\text{-}\mathrm{order}\;\mathrm{release}\;\mathrm{model}:\;F_{t}=100\times (1-e^{-k_{1}t})$$

(7) $$\mathrm{Higuchi}\;\mathrm{release}\;\mathrm{model}:\;F_{t}=K_{H}\times t^{1/2}$$

(8) $$\mathrm{Korsmeyer}\text{-}\mathrm{Peppas}\;\mathrm{release}\;\mathrm{model}:\;F_{t}=K_{P}\times t^{n}$$

(9) $$\mathrm{Weibull}\;\mathrm{model}:\;F_{t}=100\times (1-e^{-Kw\cdot t^{\beta }})$$

(10) $$\mathrm{Baker}\;\mathrm{Lonsdale}\;\mathrm{model}:\;\frac{3}{2}\left[1-{\left(1-\frac{F_{t}}{F_{\infty }}\right)}^{2/3}\right]-\frac{F_{t}}{F_{\infty }}=K\times t$$

where

F_t_—amount of active substance released at t moment,
F_0_—the initial amount of drug substance in the polymer film,
K_0_—constant of zero order release rate,
K—constant of first order release rate,
K_H_—constant of Higuchi model release rate,
K_P_—constant of Korsmeyer–Peppas model release rate,
n—exponential coefficient, an indicator of release mechanism of active substance,
K_w_—constant of Weibull model,
β—shape parameter,
F_∞_—the maximum amount of substance that can be released from the polymer film,
t—time.

The data fitting was carried out by linear or non-linear regression using Matlab 7.1. The Akaike information criterion (AIC) and the correlation coefficient R^2^ were the criteria for selecting the model that most faithfully depicted the release profile of each studied formulation. A prediction of the model that is as good as possible requires R^2^ to be as close to 1 as possible and the AIC to have minimum values [41,42,43].

#### 2.3.13. Antifungal Activity of Polymeric Films

The antifungal activity was performed against standard microorganisms (ATCC = American Type Culture Collection): *Candida albicans* ATCC 10231 and two clinical strains: *Candida albicans* 4746 and *Candida albicans* 4763.

The antifungal activity was evaluated using the disk diffusion methods (CLSI 2009) on Mueller–Hinton agar (Oxoid, Hampshire, UK) and Mueller–Hinton agar Fungi (Biolab, Lawrenceville, Georgia).

Each test microorganism was deposited on the surface of the nutrient agar and was uniformly distributed with a sterile swab. The turbidity of suspensions of standard microorganisms was adjusted to 0.5 McFarland standard, with a final turbidity of approximately 1 × 10^8^ CFU/mL. On the surface of the agar in Petri dishes, we applied the sterile discs of polymeric films, with 5 mm in diameter, and also one disc with Fluconazole—a25 µg/disk (FCA 25) for quality control. The plates were left for 10 min at room temperature to ensure equal diffusion of the compound in the medium and were then incubated at 35 °C for 24 h. All tests were carried out in triplicate. The evaluation of antibacterial and antifungal activity was performed by measuring the diameters of the inhibition zones (d, mm) [44,45].

## 3. Results and Discussion

The films were prepared by using unmodified pullulan (P) and oxidized pullulan (T-OP), specifically 6-carboxypullulan (T-OP). The films were successfully generated using a simple physical cross-linking process incorporating inter and intramolecular hydrogen bonding. The pullulan was modified by an oxidation reaction using a selective oxidizing agent, TEMPO, in order to selectively introduce carboxylic groups on the polymer chain [46]. The oxidation of the pullulan was easily confirmed by NMR spectroscopy, with the ^13^C-NMR spectra indicating the presence of a new signal that was well-defined at 176 ppm, specific to the carboxylic groups (see Table S1 and Figure S2 from Supplementary content). The Zeta potential of pullulan diminishes from the unoxidized form, which exhibits a moderately positive value of 1.5 ± 0.2 mV, to a negative value of −27.8 ± 1.6 mV, confirming the presence of a significant quantity of negatively charged groups (COO¯) in the macromolecular structure during TEMPO oxidation. Pullulan and oxidized pullulan, which has numerous hydroxyl (P) and hydroxyl/carboxyl groups (T-OP), have multiple hydrogen bonds with NaCMC, resulting in a complex hydrogen-bonding network.

### 3.1. Solubility Study

The experimental study considered increasing the solubility of TH using a non-ionic surfactant, which is gentle on the skin tissue and which would increase the permeability of the medicinal substance through the epidermis [47]. In the selection of Tween-80 concentrations, it was taken into account that they should be above the reported critical micelle concentration [48].

The experimental data are shown in Table 2 and a spectacular increase in TH solubility is observed, starting from a concentration of 2% Tween-80.

The experimental data obtained showed a linear increase in the solubility of TH with the concentration of Tween-80. At a concentration of 2% in the surfactant, the solubility of TH was increased 893.77 above the solubility of TH in water, which is only 0.0000738 g [49,50]. At a concentration of 10% in Tween-80, the solubility of TH is at its maximum and increases compared to water by 1355 times. These results are superior to other studies, which show the solubilization of TH in organic solvents, known to be skin irritants [51]. In selecting the optimal concentration of Tween-80 in which to solubilize the drug, it was taken into account that it should be around the reported critical micelle concentration [48] but, at the same time, not to exceed the recommended concentration of 5%. For this concentration, no irritations were reported for the 10-day treatment and moderate irritations were reported for the 30-day treatment [52]. Studies have shown that Tween-80 is safe and effective for all routes of administration [11], and topical application at a concentration of 10% increases water loss and improves permeability through the epidermis [13]. The experimental results obtained on TH solubilization, together with the biosafety studies reported for Tween-80, were the basis for choosing the 2% Tween-80 concentration as being effective and safe for inclusion in the bioadhesive polymeric films developed in this study.

### 3.2. Estimation of Drug Loading and Entrapment Efficiency

The entrapment efficiency (EE) and loading capacity (LC) of the drug in the polymeric films were determined by the spectrophotometric method. The wavelength at which TH absorbs in phosphate buffer is 285 nm, the range of linearity is 5–50 µg/mL, and the regression coefficient is 0.9998, a value close to unity, which indicates compliance with Beer’s law in the chosen concentration range, and the value of significance is equal to $5.43\times 10^{-13}$ [31,53]. Calculation of the amount of drug loaded was performed by using the equation Absorbance=0.0224·Concentration+0.0260. The results are presented in Table 3 and show the correlation between entrapment efficiency (EE) and loading capacity (LC) and the amount of TH per unit area of polymeric film (cm^2^). The loading efficiency results of over 95% indicate a uniform distribution of the drug in both formulations, which suggests a high compatibility between the ingredients of the polymer films. The slight increase in the loading capacity of TH in the F2_TH polymeric film is due to the carboxyl groups in the T-OP structure that bind to the polar groups of Tween-80 that has the drug incorporated into the micelles. These data are correlated with the slightly higher amount of TH in the same polymeric film. Both films have a large amount of drug per 1 cm^2^ surface area, so the treatment of fungal infections can be adapted depending on the affected skin area.

### 3.3. Structural Characterization by FTIR Spectroscopy

**Figure 1:**
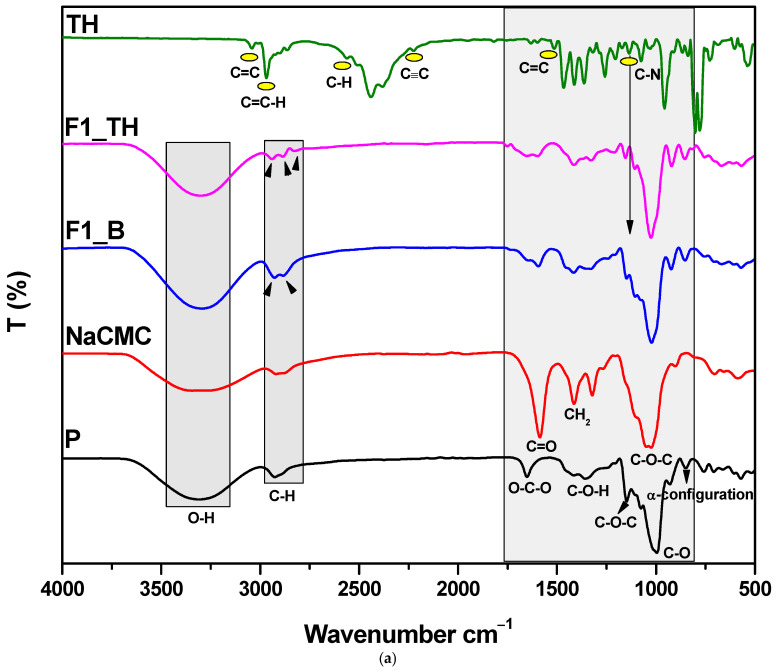

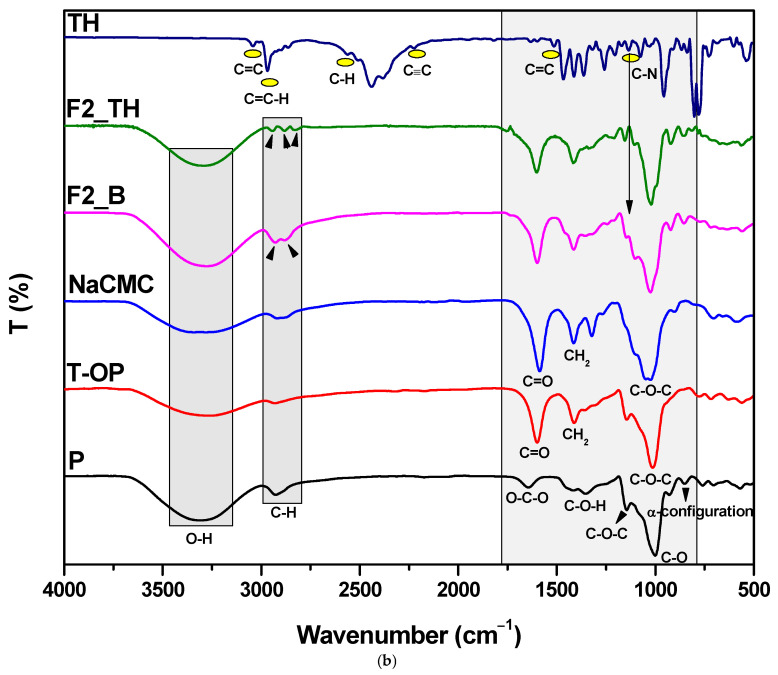
(**a**) ATR-FTIR spectra of P, NaCMC, TH, and F1_B, F1_TH composite films without and with TH; (**b**) ATR-FTIR spectra of P, T-OP, NaCMC, TH, and F2_B, F2_TH composite films without and with TH.

To confirm the changes that occurred during the film formation, FTIR spectroscopy was performed, as shown in Figure 1. Analyzing the spectra, it was observed that the pullulan has a strong and wide absorption band at around 3308 cm^−1^, which means that it has repeating units of –OH groups. Another strong absorption at 2929 cm^−1^ shows that the pullulan has an sp^3^ C–H bond corresponding to the alkane compounds. A band around 928 cm^−1^ was also observed, proving the presence of α-(1→6) and α-(1→4) linkages, respectively. The C1-4 chair conformation of pullulan is evidenced by the absorption around 846 cm^−1^ and 753 cm^−1^. The band at around 1649 cm^−1^ is specified to the valence vibrations of the C–O–C bond and the glycosidic bridge [54]. The pullulan’s α-configuration is indicated by the band at about 851 cm^−1^ [46,55]. In the case of T-OP after oxidation, an adsorption band around 1602 cm^−1^ can be attributed to the C=O stretching of free carboxylate groups, since the adsorption band at 1413 cm^−1^ represents the C-O symmetric stretching of dissociated carboxyl groups [56] (Figure S3, Supporting Information). Compared with the FTIR spectrum of pullulan, the FTIR spectrum of NaCMC is slightly different. The NaCMC has a broad band at 3305 cm^−1^ for the hydroxylic groups (-OH), a moderate band at 2918 cm^−1^ for the vibration of the methylene group (-CH_2_-), a sharp band with moderate intensity at 1587 cm^−1^ for the stretch absorption from the carbonyl groups (C=O) of glucose monomers, a band with moderate absorption at 1413 cm^−1^ for the methylene (-CH_2_-) groups, and a strong band at 1024 cm^−1^ for the ether group (C-O-C) [57]. The FTIR spectrum of pure TH shows aromatic C≡C stretching bands at 2223 cm^−1^, aromatic C-H stretching bands at 3038 cm^−1^, aromatic alkenyl C=C-H stretching bands at 2968 cm^−1^, C-N bands at 1135 cm^−1^, and aliphatic C-H stretching bands at 2563 cm^−1^ (Figure S4, Supporting Information). The maximum out-of-plane vibration associated with the benzene ring was recorded at 771 cm^−1^. A comparison study of the ATR-FTIR spectra (Figure 1) revealed that the most pronounced shifts occurred in the 3000–2700 cm^−1^ and 1200–1500 cm^−1^ regions, whereas other regions exhibited only minimal changes. In the films, a shift toward shorter wavelengths of the center of the band corresponding to the -OH groups in the 3500–3000 cm^−1^ region was seen. This demonstrated the existence of hydrogen bonds (Figure 1a) between the polymer components. All films displayed a redshift of the band at about 2968 cm^−1^, corresponding to the aliphatic C-H deformation band associated with methyl and methylene groups, towards lower wavenumbers (Figure 1b). The band corresponding to the amine group at 2447 cm^−1^ in TH was not detected in TH films. These facts confirmed that during solvent evaporation, the TH molecules found in the solution while preparing the films were dispersed in the mixture of polymer structures without forming chemical bonds [58].

The FTIR spectrum analysis led to two conclusions: the unique bands of pullulan and NaCMC were preserved in their entirety, and there was no chemical interaction between TH and the polymers. Instead, the films were obtained through a physical cross-linking process involving intermolecular and intramolecular hydrogen bonding.

### 3.4. Energy-Dispersive X-Ray (EDX)

The elemental analysis of the films was evaluated using the energy-dispersive X-ray (EDX) spectra in order to show the carbon and oxygen peaks characteristic of the films without TH. The presence of nitrogen and chlorine confirmed the loading of TH in the polymeric films [59].

**Figure 2:**
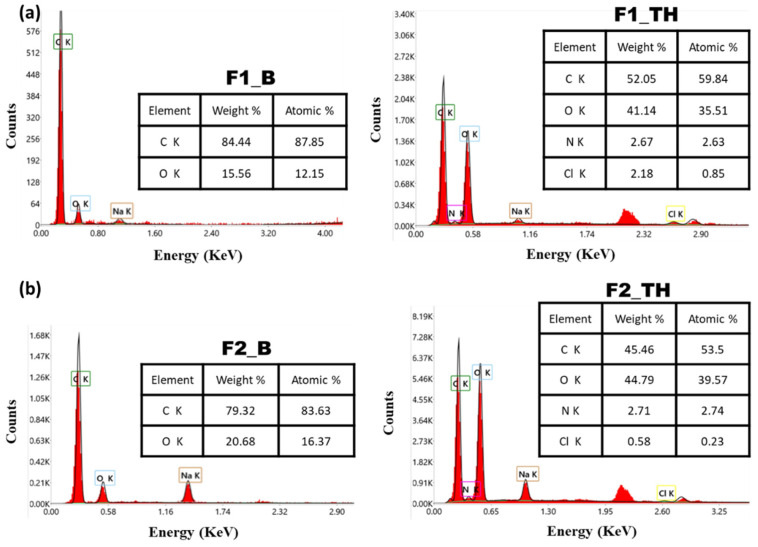
(**a**) Energy-dispersive X-ray (EDX) spectra of the composite films F1_B and F1_TH. (**b**) Energy-dispersive X-ray (EDX) spectra of the composite films F2_B and F2_TH.

The results of this determination are presented in Figure 2.

### 3.5. Mechanical Tests

The mechanical properties of the samples, such as tensile strength, elongation at break, and elastic modulus, were tested to confirm that the films have satisfactory physical performance, and they are presented in Table 4. Tensile strength indicates how well a material can withstand deformation when subjected to pressure, elongation at break shows the material’s capacity to absorb impacts, and Young’s modulus measures the rigidity of the material. The possible difference in the distribution of intra and intermolecular interactions between the polymer chains in the matrix of the P and the T-OP derivative film could have affected the mechanical properties [37,60]. The data presented in Table 4 indicate that the tensile strength of the film with P is measured at 27.30 kPa. In contrast, the tensile strength for the films with T-OP shows a decrease to 19.98 kPa, showing a weaker network structure. A decrease in elongation at break and Young’s modulus is also observed, indicating a decrease in stiffness. This reduction in stiffness could improve the processability of the films, making them more suitable for various applications where flexibility is desired.

### 3.6. Contact Angle Determination

**Figure 3:**
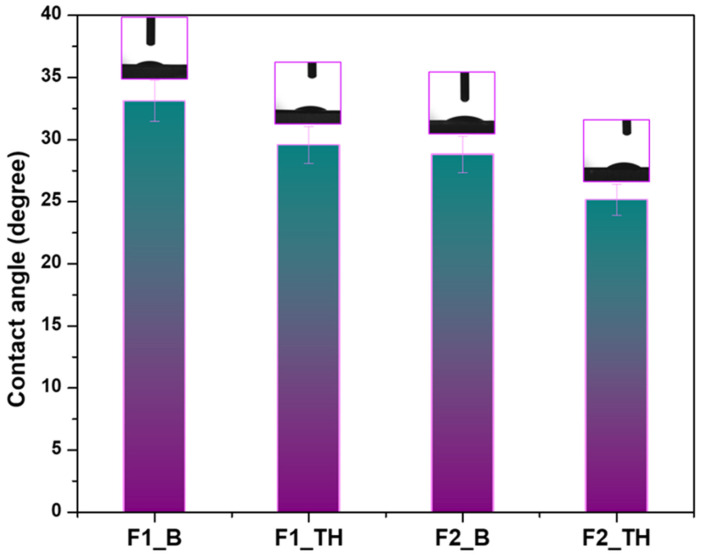
Contact angle determined with a droplet of bidistilled water on the film at ambient temperature. Mean ± SD (*n* = 10).

The hydrophilic/hydrophobic character was determined by measuring the contact angle [61]. Figure 3 illustrates the mean of 10 measurements for the contact angles of all the films. All examined films exhibit a contact angle of less than 90°, signifying hydrophilic activity on the film surface. Comparative analysis of the films F1_B and F2_B reveals a reduction in the contact angle from 33.12° ± 0.26 to 28.81° ± 0.63, indicating that the presence of carboxyl groups positively enhances the hydrophilicity of the films. The films containing TH exhibited a slight reduction in the contact angle value, keeping to the same trend [62]. The contact angle for film F2_TH was the least at 25.16° ± 0.61. The capacity of hydrophilic materials to absorb water and facilitate liquid dispersion on their surface is correlated with the efficacy of P or T-OP and NaCMC in forming hydrogen bonds.

### 3.7. Dynamic Water Vapors Sorption Measurements

**Figure 4:**
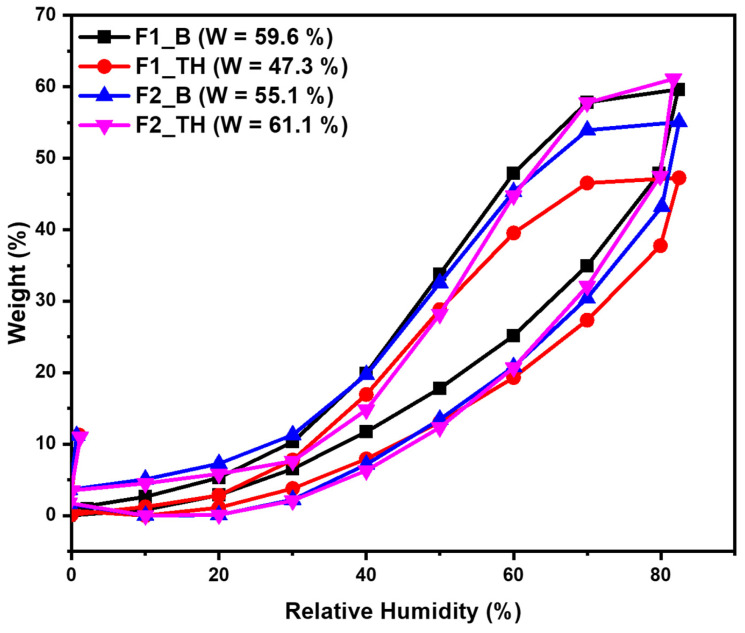
Sorption/desorption isotherms for the F1_B, F1_TH, F2_B, and F2_TH films.

The samples (F1_B, F1_TH, F2_B, and F2_TH) were also investigated by using the dynamic vapor sorption capacity. The structure of the films determines their behavior in the presence of the moisture. The water sorption/desorption behavior of the films is a crucial factor in evaluating the stability of the products, elucidating the mechanisms of water’s migration within them, and determining the mechanisms by which they gain or lose water. Thus, the water vapor sorption capacity in the dynamic regime was evaluated; the sorption/desorption isotherms are presented in Figure 4. The water sorption curves in Figure 4 match with Type IV isotherms based on the IUPAC classification [62]. This type of isotherm with hysteresis can be interpreted as being characteristic of the mesoporous adsorbents with strong affinities for water, specifically for a hydrophilic material. The size of the hysteresis is dependent on the nature of the components of the material. The polysaccharide-based network’s capacity to retain water molecules, both on hydrophilic sites and within the material’s nanopores, is the underlying cause of this hysteresis. This behavior can reflect a structural and conformational reorganization/rearrangement of the components that influence the water vapor accessibility and can eventually stop the movement of the vapors. Important information about the sample’s surface can be obtained; thus, there is reduced water vapor sorption at low values of relative humidity, RH (0 to 10%), sometimes moderate sorption at intermediate values of RH, and a sharp increase in water sorption at RH values close to 100%. The isotherms of all samples demonstrate hysteresis between sorption and desorption across the entire humidity range under investigation. In the region of low humidity, the isotherm exhibited a gradual increase, indicative of an affinity between the films and water molecules. As anticipated, the F2_TH exhibits a markedly higher water vapor sorption capacity in comparison to F1_TH. The hydrophilic character of T-OP and NaCMC, determined by the presence of hydroxyl and carboxylic groups, facilitates the increase in water sorption capacity. When F1_B is loaded with TH, electrostatic interactions and hydrogen bonds emerge, resulting in a reduction in the number of functional groups. The formation of such bonds results in a reduction in the water sorption capacity across the entire range of relative humidity.

### 3.8. Bioadhesive Properties

For local administration, the polymeric film must adhere to the skin affected by fungal infection, which has an increased humidity, and it is essential to have an adequate degree of bioadhesion; therefore, films with TH were tested (F1_TH and F2_TH). The obtained results of the adhesion measurements, expressed as the force (N), were 0.585 N for the F1_TH film and 0.645 N for the F2_TH film. In the case of F2_TH, there was a slight increase in the adhesion value, with the increase particularly being due to the presence of the hydroxylic and carboxylic groups in their molecular structure. These functional groups can establish hydrogen bonds with the polar groups present on the skin’s surface, particularly in the outermost layer of the epidermis (the stratum corneum), which contains water and various polar components (such as proteins and lipids). These bonds allow the film to adhere to the skin’s surface, providing a stable interface that enhances the delivery of the antifungal agent, TH. Furthermore, these films can form a protective layer over the skin, preventing moisture loss, promoting healing, and reducing irritation [63].

### 3.9. In Vitro Release Study

**Figure 5:**
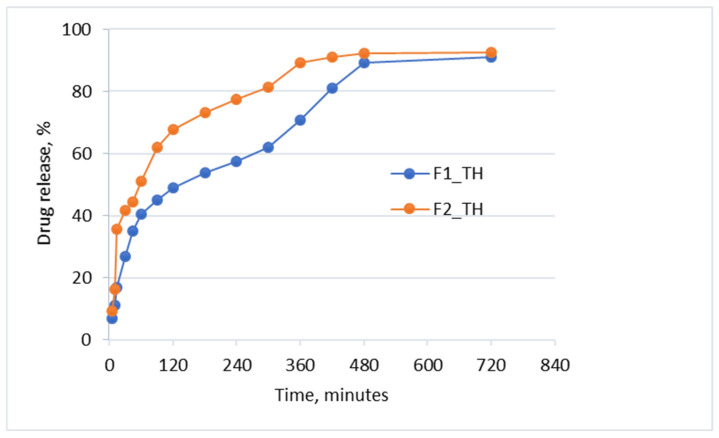
In vitro release profile for F1_TH and F2_TH.

The results of the release of TH from the polymeric films are shown in Figure 5 and are consistent with the FTIR analysis, which indicates the presence of weak inter- and intramolecular bonds, but also with the DSV and the contact angle analysis, which confirms the hydrophilic character of the polymeric films.

The polar character of the carboxyl group, which comes from the modified pullulan in the F2_TH polymeric film, increases its hydrophilicity and enables faster release of TH from the matrix. Thus, in the first 15 min, approximately 35% of the drug is released, compared to a release of 15% from the F1_TH polymeric film. After this time interval, the release of the drug is slower, being 67% for F2_TH and 49% for F1_TH at 120 min; the threshold of 80% released drug is reached after 300 min for the F2_TH matrix and 400 min for F1_TH matrix. A release of over 90% of the drug from both polymeric films occurs after 720 min (12 h), while some studies show the release of TH from Lamisil cream 1%, in the same phosphate buffer pH 7.4, to be below 70% in the same period of time [5]. The faster release of TH from the F2_TH polymeric film can be explained by the presence of the carboxyl group of pullulan, which is deprotonated at pH 7.4, resulting in relaxation of the polymeric chains, consequently releasing a larger amount of drug [5]. The release of terbinafine from both polymeric films has the advantage of reaching the therapeutic concentration immediately after administration, being followed by a delayed release, thus keeping the drug in the therapeutic window for maximum effectiveness against fungal infections [51].

### 3.10. Analysis of In Vitro Drug Release Kinetics

Mathematical models are used to identify the release profile of the drug from the polymeric film. In this paper, zero order, first order, Higuchi, Korsmeyer–Peppas, Weibull and Baker Lonsdale models were applied (Table 5). From all the studied models, the most suitable were the Korsmeyer–Peppas and Weibull models.

**Figure 6:**
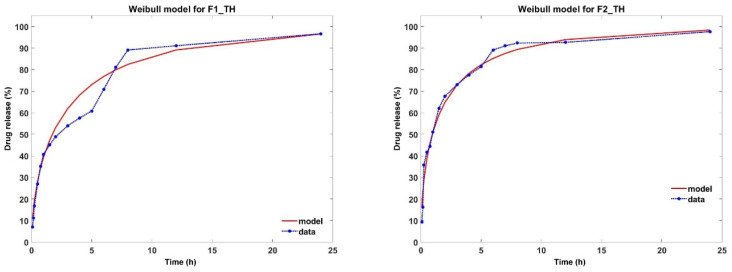
Fitting the TH release profile to the Weibull model.

In the Korsmeyer–Peppas model, ‚n’ is the diffusion exponent that describes the drug release mechanism. As shown in Table 5, the value of‚ n’ was <0.5 for all samples, indicating that TH release follows a Fickian diffusion mechanism. Although the regression coefficient (R^2^) for this model has values higher than 0.85, the AIC values are very high and unsatisfactory (Table 5) [64]. The results obtained from the release kinetics analysis highlight that TH release from the analyzed polymeric films fits best with the Weibull model, as it exhibits the most optimal control parameters, as shown in Table 5. Moreover, the values of the shape parameter β indicate that the release profile of TH from the two studied formulations follows a parabolic trend, characterized by a monotonic curve with a high initial slope, which is more pronounced than that of an exponential curve (Figure 6) [65].

**Figure 7:**
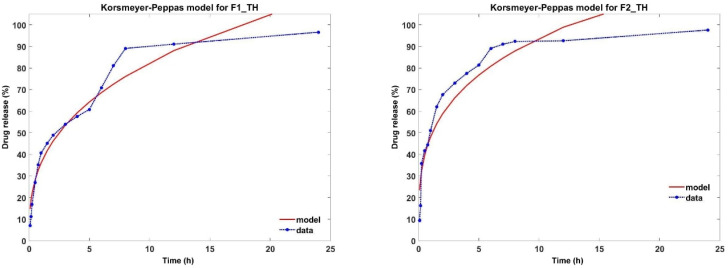
Fitting the TH release profile to the Korsmeyer–Peppas model.

Over time, the release profile of TH from different pharmaceutical forms has been associated with the Korsmeyer–Peppas model [5,8,51]. In our study, the values of n’ and R^2^, from the Korsmeyer–Peppas model, can lead us to the conclusion that the release of TH occurs according to Fickian diffusion. However, from Figure 7, one can see the limitations imposed by the Korsmeyer–Peppas model. An alternative is the Weibull model of drug release from different pharmaceutical forms [66,67]. As can be seen from Figure 6, the Weibull model fits the release profile better, having a better degree of accuracy and, moreover, this model allows us to make correlations between the characteristic parameters of the model and the morphological structure of the polymeric films. Thus, the Weibull model was able to effectively characterize the entire release process. Martín-Camacho et al. [66] conducted a comparison between the two models by analyzing 451 datasets establishing a correlation between the, n’ and, β’ parameters of the Korsmeyer–Peppas and Weibull models. The conclusion reached by the researchers is that the Weibull model describes the drug release profile independently of the characteristics of the polymer matrix, being clearly superior to the Korsmeyer–Peppas model. In our study, even though both polymeric films obey the Weibull model, it can be seen from Figure 7 that the F2_TH film containing oxidized pullulan resulted in a more pronounced drug release. We can conclude that in the case of this polymeric film, the presence of polar groups influences the drug release process. In conclusion, the release of terbinafine from the analyzed samples fits best with the Weibull model, exhibiting a release curve with a parabolic profile.

### 3.11. Antimicrobial Activity

**Figure 8:**
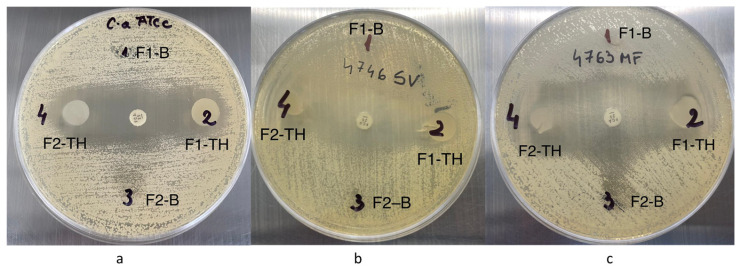
Antifungal activity of (**a**) *Candida albicans* ATCC 10231, (**b**) *Candida albicans* 4746, (**c**) *Candida albicans* 4763.

All the polymeric films were tested towards *Candida albicans* ATCC-10231 and two other clinical strains. In Table 6, information is presented about the antifungal activity in terms of the zone of inhibitions (in mm), and the results are expressed as means ± SD (Standard Deviation); the photographic images of the zone of inhibition are shown in Figure 8.

The polymeric films loaded with TH show relatively good antimicrobial activity against *Candida albicans* ATCC 10231 and those two clinical strains: *Candida albicans* 4746 and *Candida albicans* 4763. In the tests performed, it was found that the diameter of the inhibition zone of the polymeric films loaded with TH (F1_TH and F2_TH) increases with the slight increase in the concentration of the drug in the samples. At the same time, the inhibition of the development of microorganisms is stopped, thanks to the new functional groups of the carboxyl type in the structure of pullulan (F2_TH). This activity is found in the three *Candida albicans* species studied, and the antifungal activity of the TH-loaded polymeric films is slightly higher for the isolated species.

The charge of these functional groups is correlated with a negative Zeta potential, which is higher for carboxyl groups, which facilitates the destruction of the cell membrane and finally the death of the bacterial cells [68]. For the F2_TH sample, we can speak of a synergism that is due on the one hand to the slightly higher concentration of TH in the sample and on the other hand to the carboxyl groups, which together determine the decrease in the concentration of ergosterol, a phenomenon that leads to the death of fungal cells [69]. Our antifungal study results are comparable to Kraisit, P et al., who prepared fluconazole (FZ)-loaded solid lipid nanoparticles loaded in films with chitosan by the film casting method [17]. Taken together, these results suggest that polymer films containing TH as an active drug can be promising for the treatment fungal infection.

## 4. Conclusions

Polymeric films are safe systems for delivering drugs to the target site, especially for dermatophytoses that affect the skin. Terbinafine, an antifungal used in treating superficial fungal infections, has a bioavailability of only 40%. To increase its solubility, in our study the drug was solubilized with Tween-80 at a concentration of 2%. This treatment increased terbinafine’s solubility almost 900 times compared to water. The prepared films are able to interact with skin lipids, preventing drug penetration and cell destruction. The carboxyl group from the oxidized pullulan in the F2_TH film further enhances the antifungal effect. The films have a hydrophilic character and a type IV isotherm, which is specific to hydrophilic mesoporous materials. The drug-loaded films have a loading efficiency of over 95%, indicating good dispersion of terbinafine over the entire surface. The drug concentration of over 1.55 mg/cm^2^ helps control the drug administration dose. The films also have a rapid release in the first 15 min after administration, followed by a delayed release for 400 min, increasing patient compliance.

In conclusion, while advancements in polymeric films for skin drug discovery have been significant, there are still challenges to address. The continued development of effective polymeric films based on natural polymers could offer innovative solutions for skin infections. As technology evolves, it is crucial for both scientists and clinicians to adopt these films to explore new opportunities and enhance current treatments for skin diseases.

## Figures and Tables

**Table 1:** Composition of the films prepared in this study.

Samples	Tween 80 (g)	2% Tween 80 Solution of TH (g)	P (g)	T-OP (g)	NaCMC (g)	Glycerol (g)
F1_B	10	-	0.4	-	0.16	0.4
F1_TH	-	10	0.4	-	0.16	0.4
F2_B	10	-	-	0.4	0.16	0.4
F2_TH	-	10	-	0.4	0.16	0.4

**Table 2:** The solubility of TH in water and in solution of Tween-80.

Concentration of Tween-80 g%	Solubility (g% ± SD)	Increasing Solubility Toward Water ± SD
Water (0% Tween-80)	7.38 × 10^−5^	-
2	0.6596 ± 2.08	893.77 ± 2.17
4	0.8133 ± 1.95	1102.03 ± 2.06
6	0.8653 ± 1.67	1172.49 ± 1.98
8	0.9444 ± 1.14	1279.67 ± 1.53
10	1 ± 0.87	1355.01 ± 1.12

**Table 3:** The loading capacity (LC) and entrapment efficiency of polymeric films.

Polymeric Films	LC ± SD%	EE ± SD%	m_TH_/cm^2^ mg/cm^2^
F1_TH	5.95 ± 0.006	96.40 ± 0.04	1.55
F2_TH	6.04 ± 0.005	95.25 ±0.03	1.83

**Table 4:** Mechanical properties of composites films.

Sample Code	Tensile Strength (kPa)	Young’s Modulus (kPa)	Elongation at Break (%)
F1_B	27.30	5.77	489.95
F2_B	19.98	5.70	272.51

**Table 5:** Data fitting results of in vitro terbinafine release profile from polymeric films.

Kinetic Model	Parameters	Sample	
		F1_TH	F2_TH
Zero order	K_0_	3.748	3.202
	R^2^	0.658	0.498
	AIC	138.420	144.043
First order	K	0.145	0.154
	R^2^	0.915	0.839
	AIC	6.887	20.342
Higuchi	K_H_	26.613	30.568
	R^2^	0.883	0.775
	AIC	126.649	144.365
Korsmeyer–Peppas	K_P_	35.991	48.097
	n	0.360	0.290
	R^2^	0.938	0.894
	AIC	108.589	118.123
Weibull	K_W_	0.499	0.713
	β	0.600	0.550
	R^2^	0.950	0.970
	AIC	−5.150	−7.252
Baker Lonsdale	K_B_	0.019	0.023
	R^2^	0.885	0.775
	AIC	−54.089	−33.577

**Table 6:** In vitro antifungal studies.

Sample/Quality Control	Diameter of Inhibition Zones (d, mm)			
	Concentration of Drug mg/Sample	*C. albicans* ATCC 10231	*C. albicans* 4746	*C. albicans* 4763
F1_B	-	0	0	0
F2_B	-	0	0	0
F1_TH	1.26	17.0 ± 0.00	16.0 ± 0.00	20.0 ± 0.00
F2_TH	1.44	20.1 ± 0.05	21.1 ± 0.05	21 ± 0.01
FCA (25 µg/disc)	-	25.1 ± 0.05	25.7 ± 0.06	25.7 ± 0.06

## Data Availability

The original contributions presented in this study are included in the article/Supplementary Material. Further inquiries can be directed to the corresponding author.

## Supplementary Materials

The following supporting information can be downloaded at: https://www.mdpi.com/article/10.3390/polym17081004/s1, Figure S1. The aspect of the films without (A) and with TH (B); Table S1. The specific signals of each carbon atom in the pullulan structure, resulting from the ^13^C-NMR spectra of the unoxidized (P) and oxidized pullulan (T-OP) sample; Figure S2. ^13^C-NMR/D_2_O (top) and ^1^H-NMR/D_2_O (down) spectra of the pullulan before and after oxidation; Figure S3. FTIR spectra of the of pullulan before (P) and after oxidation (T-OP); Figure S4. FTIR spectra of the terbinafine hydrochlorite (TH).
