# Supplementary material for: Development and Characterization of Polymeric Films Loaded with Terbinafine for Fungal Infection Treatment
Source: Polymers (Basel). 2025 Apr 8;17(8):1004. doi: 10.3390/polym17081004 (PMC12030444; doi:10.3390/polym17081004)
Supplement: Supplementary file 1 [file polymers-17-01004-s001.zip › polymers-3510900-supplementary.pdf]

Supporting Information

Development and characterization of polymeric films loaded with terbinafine for fungal infection treatment

Gabriela Biliuta[1], Simona Petronela Gherman[2,*], Raluca Ioana Baron[1], Alexandra Bargan[3], Lăcrămioara Ochiuz[2], Cristina Gabriela Tuchilus[4], Adrian Florin Șpac[2], Daniela Elena Zavastin[2]

[1] Polyaddition and Photochemistry Department, "Petru Poni" Institute of Macromolecular Chemistry of Romanian Academy, 41A Gr. Ghica-Voda Alley, 700487 Iasi, Romania

[2] Faculty of Pharmacy, "Grigore T. Popa" University of Medicine and Pharmacy Iasi, 16th University Str., 700115 Iasi, Romania

[3] Department of Inorganic Polymers, "Petru Poni" Institute of Macromolecular Chemistry of Romanian Academy, 41A Gr. Ghica-Voda Alley, 700487 Iasi, Romania

[4] Faculty of Medicine, "Grigore T. Popa" University of Medicine and Pharmacy Iasi, 16th University Str., 700115 Iasi, Romania

* Author to whom correspondence should be addressed.
E-mail: simona.gherman@umfiasi.ro (S.P.G.)

The aspect of the films

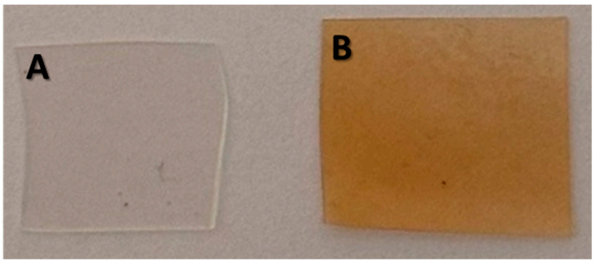

Figure S1. The aspect of the films without (A) and with TH (B)

Nuclear magnetic resonance spectroscopy (NMR)

For NMR analysis, samples were prepared by dissolving 10 mg (for ^{1}H NMR) and 50 mg (for ^{13}C NMR) of polymer in 0.7 mL of D_2O. The NMR spectra were obtained on a Bruker Advance DRX 400 MHz Spectrometer, equipped with a 5 mm QNP direct detection probe and z-gradients.

Table S1. The specific signals of each carbon atom in the pullulan structure, resulting from the ^{13}C-NMR spectra of the unoxidized (P) and oxidized pullulan (T-OP) sample.

**Sample**	**C_1 (ppm)**	**C_4 (ppm)**	**$C_{2,3,5}$ (ppm)**	**C_6-6g (ppm)**	**C_6-4g (ppm)**	**COOH (ppm)**
**P**	101-103	80	72-76	69	63	-
**T-OP**	97-98	76	69-71	65	57	**175**

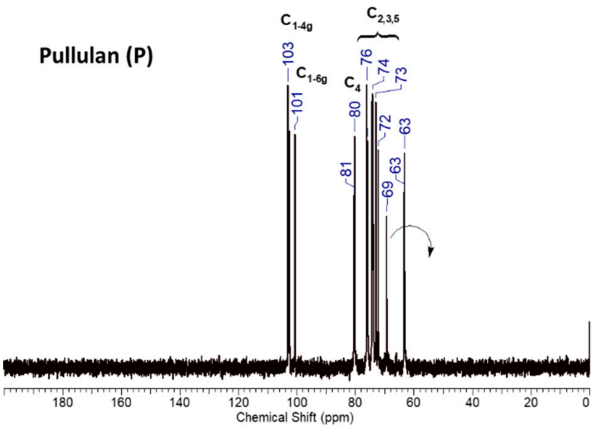

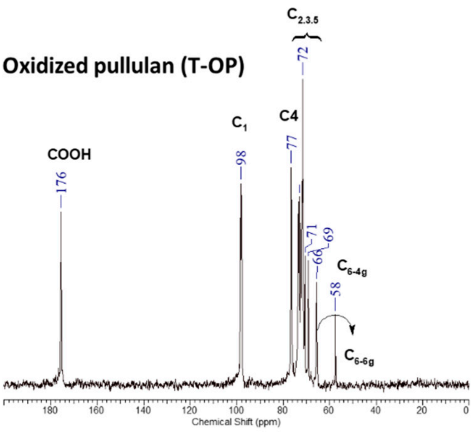

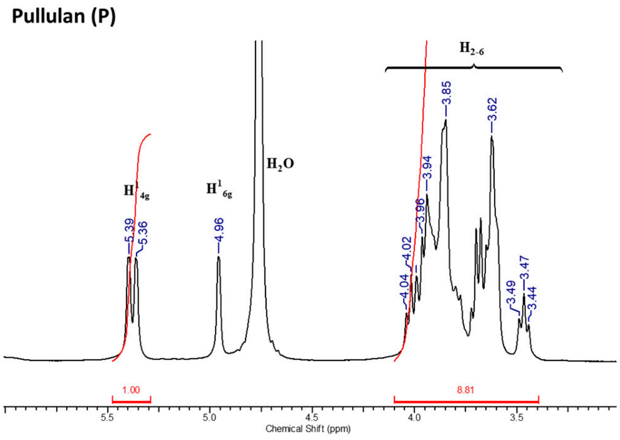

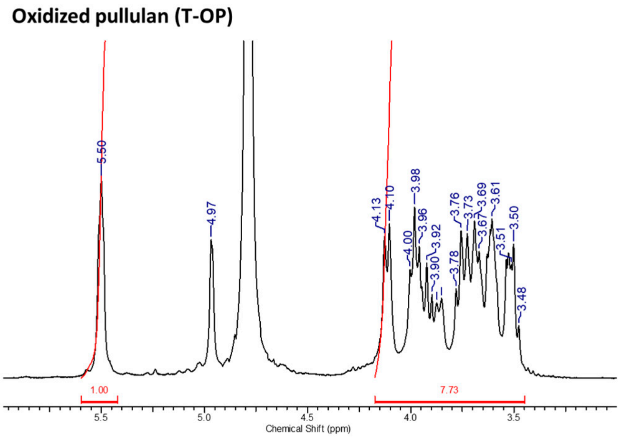

Figure S2. ^{13}C-NMR/D_2O (top) and ^{1}H-NMR/D_2O (down) spectra of the pullulan before and after oxidation.

Structural characterization by FTIR spectroscopy

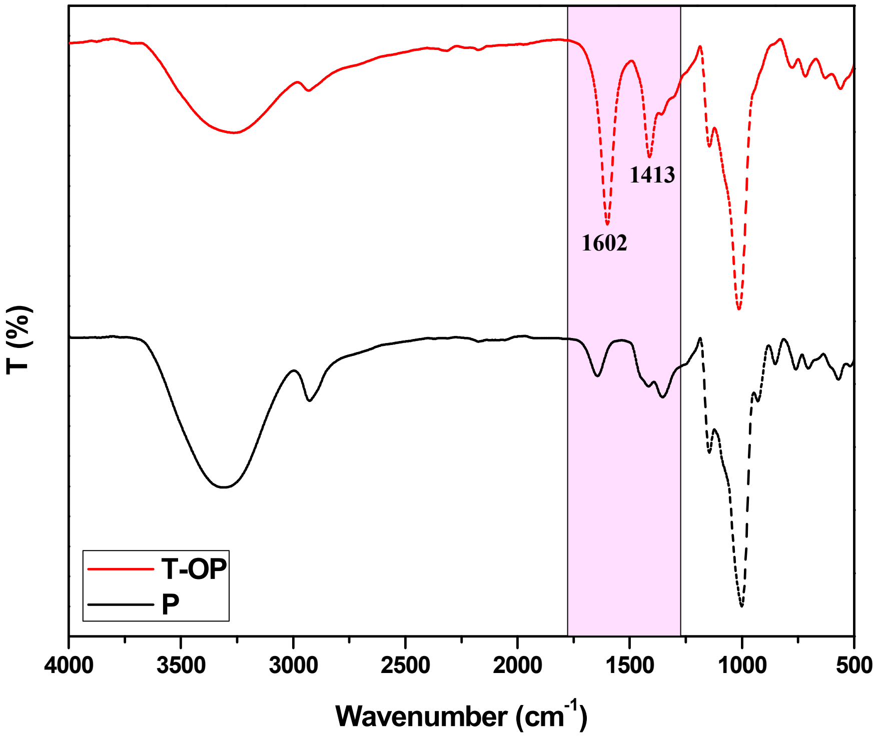

Figure S3. FTIR spectra of the of pullulan before (P) and after oxidation (T-OP).

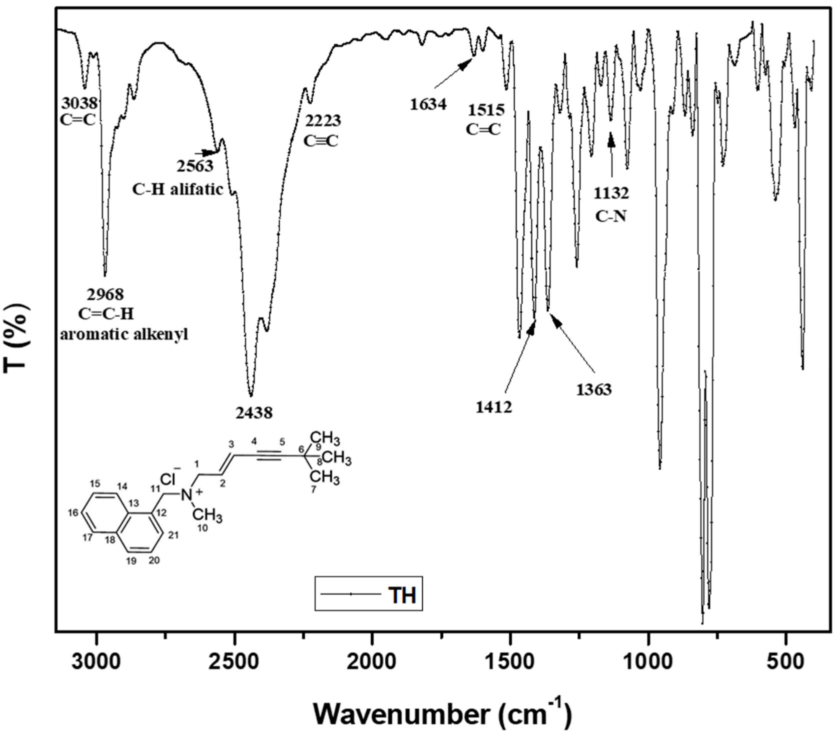

Figure S4. FTIR spectra of the terbinafine hydrochlorite (TH).
